# Late Type 3b Endoleak with an Endurant Endograft

**DOI:** 10.1155/2015/783468

**Published:** 2015-12-20

**Authors:** Mehmet Barburoglu, Bulent Acunas, Yilmaz Onal, Murat Ugurlucan, Omer Ali Sayin, Ufuk Alpagut

**Affiliations:** ^1^Istanbul Medical Faculty, Istanbul University, Department of Radiology, Millet Caddesi, Capa, Fatih, 34390 Istanbul, Turkey; ^2^Istanbul Medical Faculty, Istanbul University, Department of Cardiovascular Surgery, Istanbul, Turkey

## Abstract

Endovascular stent grafting with different commercially available stent graft systems is widely applied for the treatment of abdominal aortic aneurysms with high success rates in the current era. Various types of endoleaks are potential complications of the procedure. They usually occur in the early period. In this report, we present type 3b endoleak occurring 14 months after a successful endovascular abdominal aortic aneurysm repair with a Medtronic Endurant stent graft.

## 1. Introduction

Endovascular abdominal aortic aneurysm repair (EVAR) is a safe and efficient method, which has been widely used for treatment at abdominal aortic aneurysms (AAA). New generation endografts make it possible to treat more complex AAA with less perioperative mortality and systemic complications [1–4]. However, endoleak is still a common problem of EVAR that is characterized by persistent blood flow within the aneurysm sac [5, 6]. White et al. classified endoleaks into four types and type 3b endoleak originates from a defect in the graft fabric [7].

We report a case of late type 3b endoleak from a tear in the main body of an Endurant (Medtronic Endovascular, Santa Rosa, Calif., USA) endograft 14 months after deployment and treatment with coil embolization. Consent from the patient and his family for possible academic activities including publication acts regarding his medical history was obtained. To the best of our knowledge, this is the first report of a late type 3b endoleak with an Endurant endograft.

## 2. Case Report

**Figure 1:**
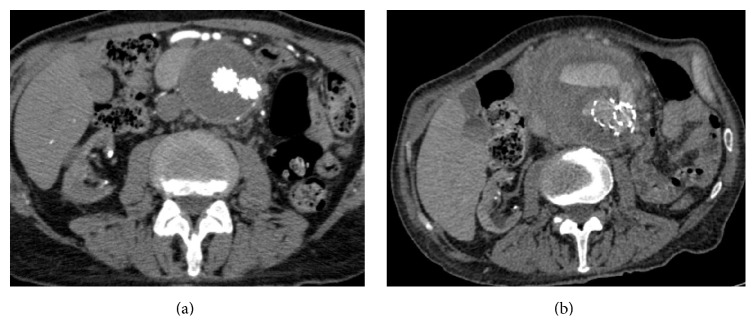
(a) 12-month follow-up CT scan indicating complete exclusion of the aneurysm with sac shrinkage after the initial procedure. (b) CT angiography showing enlarged AAA with hematoma and endoleak originating from distal main body of the EVAR graft.

An 81-year-old man underwent EVAR for the treatment of a 70 mm AAA with deployment of 36 × 16 × 166 mm main body Endurant bifurcated endograft, 16 × 20 × 120 mm contralateral limb, and extensions of 16 × 16 × 95 mm to the right and to 20 × 20 × 80 mm to the left. His medical history included heart failure with an ejection fraction of 25%, hypertension, chronic obstructive pulmonary disease, and chronic renal failure requiring hemodialysis. He has been followed up regularly with Doppler ultrasonography and computerized tomography (CT) angiography at 3, 6, and 12 months showed successful exclusion of the aneurysm sac with no leak. Bilateral internal iliac arteries were patent (Figures 1(a) and 1(b)).

**Figure 2:**
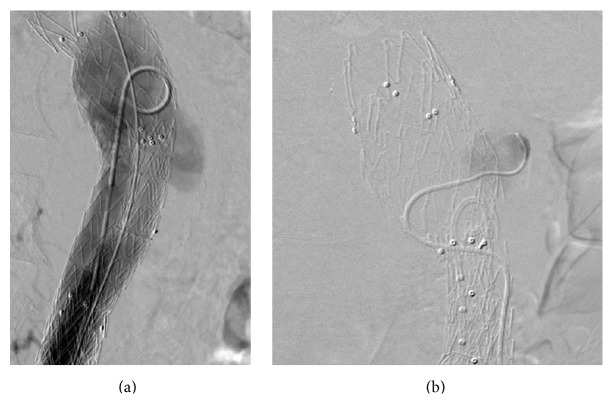
(a) Angiogram showed endoleak at the level of the bifurcation endograft. (b) Direct catheterization of the aneurysm sac through the tear of the graft.

About 14 months after EVAR, the patient was admitted to the emergency clinic with sudden onset back pain. Clinical examination revealed a pulsatile mass at the epigastrium. Computerized tomography showed enlarged AAA bounded with hematoma and an endoleak originating from distal main body of the EVAR graft (Figure 2(a)).

The patient was taken to the angiography unit in the emergency night conditions. Several angiography runs were performed to determine the location of the endoleak; type 1a endoleak was excluded. The pigtail catheter was pulled into the main body. Angiogram showed endoleak at the level of the bifurcation endograft (Figure 2(b)). This time a type 3a endoleak (disconnection of main graft components) was suspected and 16 × 20 × 120 mm stent graft was placed from the contralateral limb. Control angiogram showed persistent endoleak; however, this time a type 3b endoleak was suspected.

We discussed the treatment options with the cardiovascular surgeons and we decided to treat the patient endovascularly because of the high mortality risk of the surgery. Endovascular options for the treatment included placement of a new aorto-uni-iliac or bi-iliac stent graft or coil embolization of the sac through translumbar or transarterial approach. We decided to deploy an aorto-uni-iliac stent graft, as there was not enough distance to deploy an aorto-bi-iliac graft between the bifurcation of the previous endograft and ostia of the renal arteries. In addition, we lacked enough and appropriate sized coils for the embolization of the sac in the night conditions. We deployed an Endurant uni-iliac stent graft with a body diameter of 36 mm (36 × 14 × 105 mm, Endurant, Medtronic Endovascular, Santa Rosa, Calif., USA). The control angiogram showed persistent endoleak despite additional ballooning. The antegrade flow was still present into the right limb decoding incomplete apposition of the latter stent graft to the previous one. The patient was clinically stable and there was no marked filling into the aneurysm sac so we decided to end the procedure and evaluate the filling of the right limb with a postoperative CT examination.

The second day CT angiography showed inaccurate apposition of the new stent graft into the previous stent graft because of the inadequate diameter of the aorto-uni-iliac stent graft; 36 mm is the largest diameter in the market. The gap between the stent grafts allowed persistent blood flow into the right limb and into the aneurysm sac through the fabric tear.

**Figure 3:**
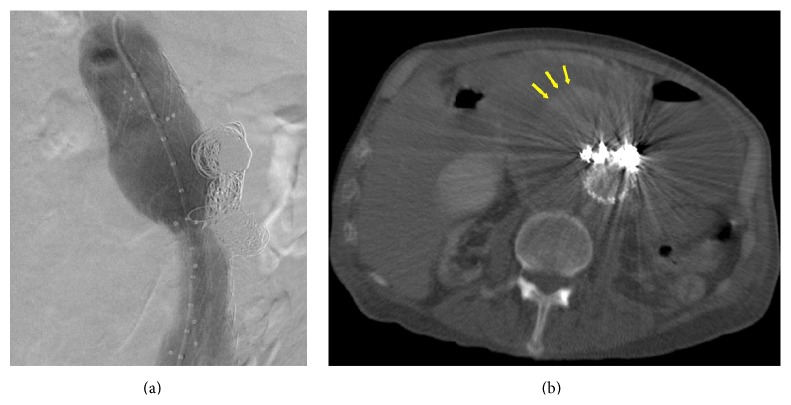
(a) Angiogram showed the complete disappearance of type 3b endoleak. (b) CT angiography showing peripheral high-density area in the sac, which could be related to fresh clot or residual contrast media during coils embolization.

We decided to coil-embolize the fabric tear and aneurysm sac through the gap between the two-stent grafts after providing the appropriate coils sizes. The gap was catheterized with 5 F vertebral catheter rather than a 2.7 F microcatheter inserted into the aneurysm sac through the fabric tear (Figure 2(b)). We coiled the aneurismal sac with detachable coils until the angiography showed the complete disappearance of type 3b endoleak (Figure 3(a)). Control CT examination denoted no contrast filling into the sac; there was peripheral high-density area in the sac, which could be related to fresh clot or residual contrast media during coils embolization (Figure 3(b)). Doppler ultrasonography also confirmed no filling in the sac.

The procedure was terminated and the patient was transferred to the intensive care unit. Unfortunately, the patient was lost due to severe heart failure 72 hours after the second procedure.

## 3. Discussion

Type 3 endoleak is a rare complication but potentially has a high risk of aneurismal rupture and always warrants urgent intervention [8]. Type 3 endoleak divides into two types: type 3a originates from disconnection of the graft compounds and type 3b originates from fabric tear [7]. In our patient, the endoleak was type 3b confirmed with direct sac catheterization through the defect with a microcatheter. We did not know the reason for the fabric defect after 14 months after the deployment. In the literature, there are reports about type 3b endoleaks with other aortic stent graft devices [9–11] but there was only one case report about early type 3b endoleak associated with Endurant endograft [12]. Our case is the first case of a late type 3b endoleak reported with an Endurant stent graft. Medtronic was informed of the case and provided no explanation.

Type 3 endoleak can be treated endovascularly or surgically. Endovascular options include repairing the defect with aortic cuff extension or placement of a new aorto-bi-iliac graft or placement of an aorto-uni-iliac graft with cross-femoral bypass. Our patient came with ruptured aneurysm and he was unstable clinically. Then we evaluated his CT angiogram; we suggested a type 3a endoleak from contralateral leg disconnection. Later type 3b endoleak from a fabric tear was proved.

We tried to close the defect with an aorto-uni-iliac graft but there was bad apposition between the new and the previous grafts despite several balloon dilatations. Therefore, we closed the endoleak with coiling the aneurismal sac through the defect. However, the patient died, 3 days after the procedure.

In conclusion, type 3b endoleak can occur on new generation endografts and it is associated with high-risk aneurysm rupture morbidity and mortality. It is difficult to diagnose the type of the endoleak only with CT angiogram without catheter angiography.
